# Protective Effect of Dietary Xylitol on Influenza A Virus Infection

**DOI:** 10.1371/journal.pone.0084633

**Published:** 2014-01-02

**Authors:** Sun Young Yin, Hyoung Jin Kim, Hong-Jin Kim

**Affiliations:** Laboratory of Virology, College of Pharmacy, Chung-Ang University, Dongjak-Gu, Seoul, South Korea; University of Cincinnati School of Medicine, United States of America

## Abstract

Xylitol has been used as a substitute for sugar to prevent cavity-causing bacteria, and most studies have focused on its benefits in dental care. Meanwhile, the constituents of red ginseng (RG) are known to be effective in ameliorating the symptoms of influenza virus infection when they are administered orally for 14 days. In this study, we investigated the effect of dietary xylitol on influenza A virus infection (H1N1). We designed regimens containing various fractions of RG (RGs: whole extract, water soluble fraction, saponin and polysaccharide) and xylitol, and combination of xylitol with the RG fractions. Mice received the various combinations orally for 5 days prior to lethal influenza A virus infection. Almost all the mice died post challenge when xylitol or RGs were administered separately. Survival was markedly enhanced when xylitol was administered along with RGs, pointing to a synergistic effect. The effect of xylitol plus RG fractions increased with increasing dose of xylitol. Moreover, dietary xylitol along with the RG water soluble fraction significantly reduced lung virus titers after infection. Therefore, we suggest that dietary xylitol is effective in ameliorating influenza-induced symptoms when it is administered with RG fractions, and this protective effect of xylitol should be considered in relation to other diseases.

## Introduction

Influenza virus is regarded as an important human pathogen because it can spread rapidly by aerosol transmission, and cause massive mortality. It is estimated that the flu pandemics in 1918–1919 (Spanish flu) and 1957–1958 (Asian flu) resulted in 20–100 million and 1–1.5 million deaths worldwide, respectively [1], [2]. The recent Mexican flu pandemic in 2009 is estimated to have resulted in 0.2 million death worldwide [1], [3]. Human influenza viruses are RNA viruses belonging to the Orthomyxoviridae, and are subdivided into types A, B and C [4]. Infections with influenza virus types B and C are restricted to humans whereas type A can also infect swine, horses and birds [5]. Mutations of influenza A virus that allow it to move from one species to another confer great virulence on the virus, which is potentially fatal to human [5]. Influenza A viruses have been the main cause of the massive mortalities suffered, and are a constant threat because of their ability to mutate.

It is clear that the most effective measure is preventing infection by the influenza virus. Although vaccination has been used for this purpose, it can only be protective when the prevalent strain matches strains contained in the vaccine [6]. Moreover, several factors including the age and health of recipients can affect vaccine efficacy [7]. Vaccine efficacy in people over 65 years of age is only 17–53%, and the main cause of death of such older individuals is influenza virus infection [8], [9]. Therefore, alternative strategies and improvements in vaccines are high priorities.

*Panax ginseng* is one of the best-known herbal treatments for promoting physical health and immune function. Previous studies have suggested that components of ginseng can act as inhibitors of influenza virus [10], [11]. We also found that the Korean red ginseng (RG) polysaccharide, saponin, and total extract were effective in reducing flu symptoms when orally administered to mice for 14 days prior to infection [12]. However, the RG extracts were not effective when given for only 5 days.

Xylitol has been used as a sugar substitute in Finland since the 1960s [13]. It is a polyalcohol, formula (CHOH)_3_(CH_2_OH)_2_., which is obtained from xylan extracted from hardwood [14]. Because cavity-causing bacteria such as *Streptococcus mutans* cannot use xylitol as an energy source [15] chewing-gum containing xylitol has been used to prevent tooth decay [16]. Studies since the early 1970s have mainly focused on the function of xylitol in dental care. In this work, we, for the first time, investigated the effect of dietary xylitol on influenza virus infection.

Much effort has been put into identifying agents that prevent influenza virus infection, but with little success. Most agents require long-term dietary intake or provide only local protection. We show that dietary intake of xylitol along with RG, or fractions of RG (referred to jointly as RGs), can provide protection against influenza virus and substantially reduce influenza virus symptoms when administered orally for just 5 days.

## Results

### The Effect of Dietary Xylitol in Combination with RGs on Lethal Influenza A Virus Infection

**Figure 1:**
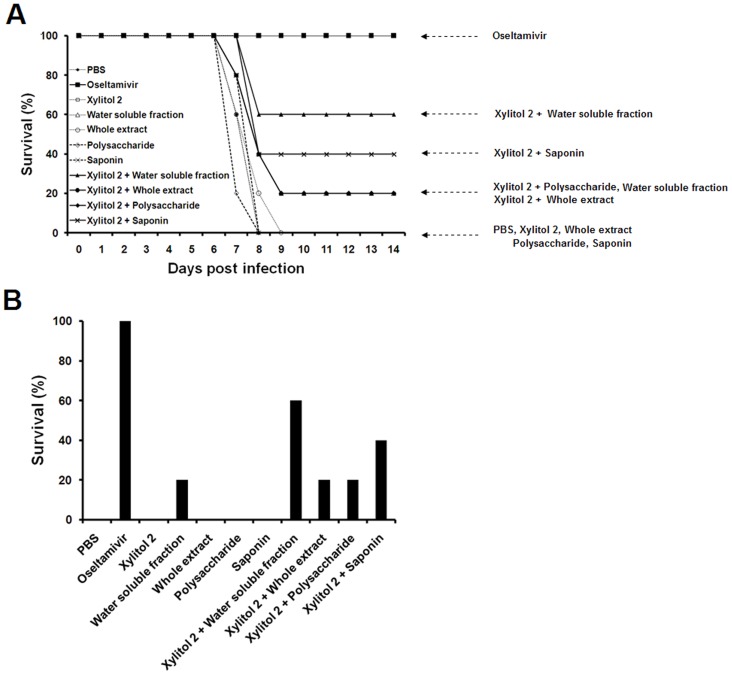
Survival of mice receiving RGs, xylitol or xylitol combined with RGs orally following lethal influenza A virus challenge. Panel A shows survival monitored for 14_50_ of influenza A virus. Panel B shows survival on the 14^th^ day post virus challenge. Mice received each regimen for 5 days prior to virus challenge and 3 days post challenge. All mouse groups except the oseltamivir group, n = 5; oseltamivir group, n = 3.

Treatment regimens used are presented in Table 1. To investigate the effect of dietary xylitol, RGs and xylitol plus RGs on lethal influenza virus infection, xylitol regimen 2 (33 mg/kg/day) was applied. Mice received each combination orally for 5 days prior to influenza A virus challenge. The oseltamivir is a neuraminidase inhibitor of influenza A and B virus [17]. The oseltamivir group was designed to be positive controls that have resistance to influenza A virus infection. All the mice receiving xylitol, RG whole extract, RG saponin or RG polysaccharide on their own died following challenge with 2X LD_50_ of influenza A virus (Fig. 1A and B). 20% of mice receiving the water soluble fraction of RG survived. The survival of mice receiving xylitol along with RGs was higher (Fig. 1A and B). Survival was in the order xylitol 2+ water soluble fraction (60%), xylitol 2+ saponin (40%), xylitol 2+ polysaccharide (20%) and xylitol 2+ whole extract (20%). These results point to a synergistic effect of xylitol and RGs on survival.

**Table 1:** Treatment regimens used in the present study.

No.	Mouse group	Prior to virus challenge(for 5 days)	Post virus challenge(for 3 days)
1	PBS	200 μl/day	200 μl/day
2	Oseltamivir	200 μl/day (PBS)^a^	10 mg/kg/day (for 7 days)^b^
3	Xylitol 1	3.3 mg/kg/day	3.3 mg/kg/day
4	Xylitol 2	33 mg/kg/day	33 mg/kg/day
5	Water soluble fraction	0.25 mg/kg/day	0.25 mg/kg/day
6	Whole extract	0.25 mg/kg/day	0.25 mg/kg/day
7	Polysaccharide	0.25 mg/kg/day	0.25 mg/kg/day
8	Saponin	0.25 mg/kg/day	0.25 mg/kg/day
9	Xylitol 1+Water soluble fraction	3.3 mg/kg/day+0.25 mg/kg/day	3.3 mg/kg/day+0.25 mg/kg/day
10	Xylitol 2+Water soluble fraction	33 mg/kg/day+0.25 mg/kg/day	33 mg/kg/day+0.25 mg/kg/day
11	Xylitol 2+Whole extract	33 mg/kg/day+0.25 mg/kg/day	33 mg/kg/day+0.25 mg/kg/day
12	Xylitol 2+Polysaccharide	33 mg/kg/day+0.25 mg/kg/day	33 mg/kg/day+0.25 mg/kg/day
13	Xylitol 2+Saponin	33 mg/kg/day+0.25 mg/kg/day	33 mg/kg/day+0.25 mg/kg/day

Mice received PBS, xylitol and/or RGs orally for 5 days prior to virus challenge and for 3 days post virus challenge.

^a^ The oseltamivir group received PBS orally for 5 days prior to virus challenge.

**Figure 2:**
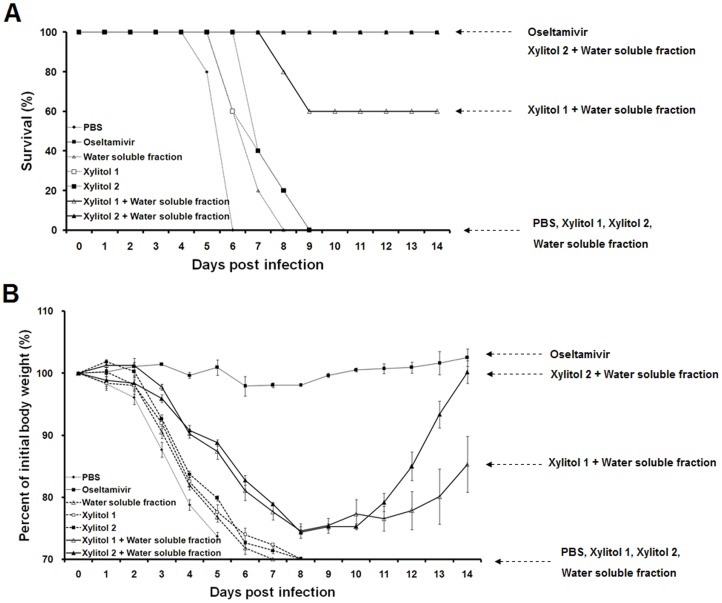
Effect of dose of xylitol on lethal influenza A virus infection. Mice were challenged with 2X LD_50_ of influenza A virus. A and B show survival rates and changes in body weight, respectively. Details as in the legend to Fig. 1. Body weights one day before virus challenge were set at 100%. Data are means ± SEMs of body weights of mice that survived. All mouse groups except the oseltamivir group, n = 5; oseltamivir group, n = 3.

**Figure 3:**
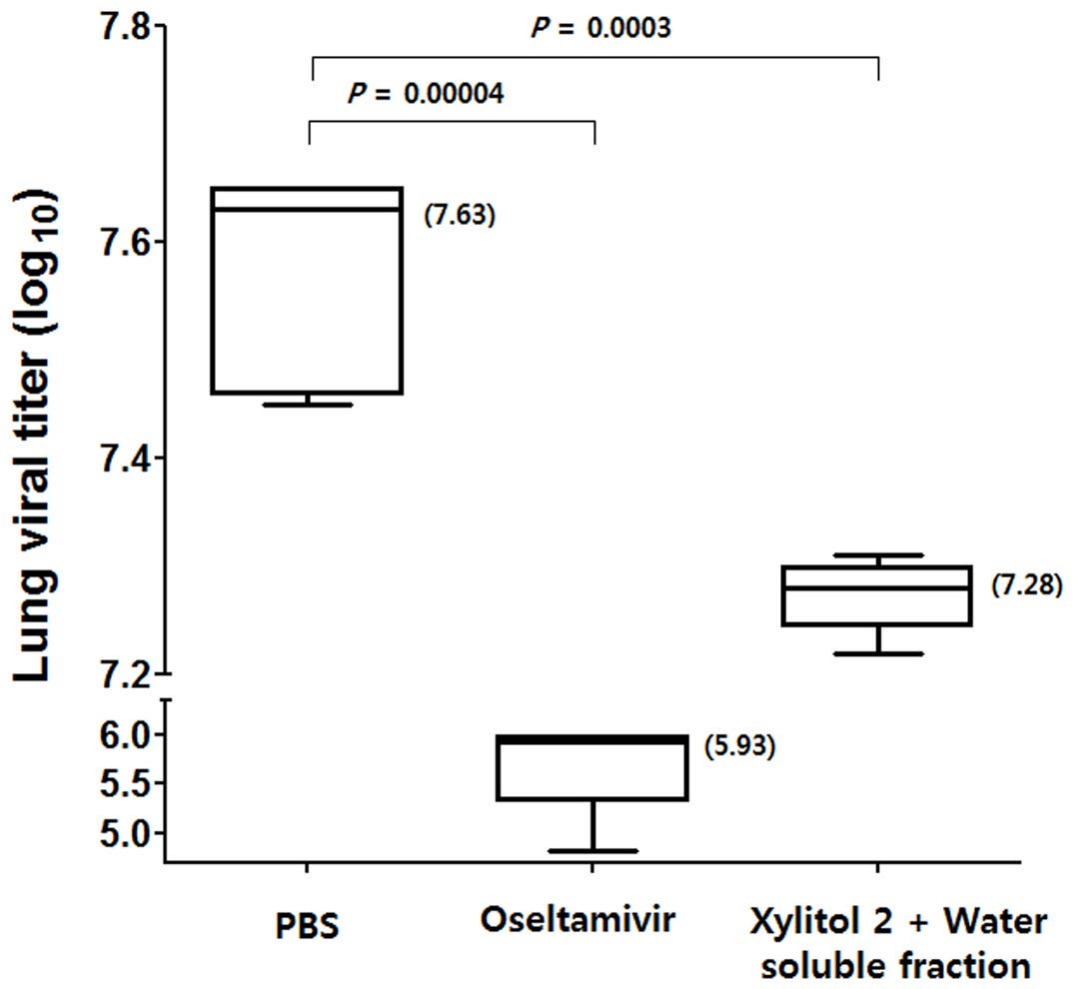
Virus titers in mouse lungs receiving PBS, oseltamivir and xylitol 2 with water soluble fraction following lethal influenza A virus challenge. For experimental details see Fig. 1 legend and Materials and Methods. Mice were challenged with 2X LD_50_ of influenza A virus. The center line of the box represents the median, and the top (Q3) and bottom (Q1), the 75^th^ and 25^th^ percentiles, respectively. The values are plaque forming units (PFUs). The top and bottom whiskers represent outliers. The numbers in parenthesis are median values. PBS, n = 5; oseltamivir, n = 5; xylitol 2+ water soluble fraction, n = 5.

^b^ The oseltamivir group received oseltamivir orally for 7 days post virus challenge while other groups received the appropriate regimen for 3 days. These regimens were used for the experiments of Figs. 1, 2 and 3.

### Protection as a Function of Dosage of Xylitol

We examined the effect on survival of the dosage of xylitol in combination with the water soluble fraction of RG. We used two doses of xylitol, xylitol 1 (3.3 mg/kg/day) and xylitol 2 (33 mg/kg/day). As shown in Fig. 2A, all mice receiving xylitol 1, xylitol 2 or the water soluble RG fraction on their own died, whereas 100 and 60% of mice survived in response to xylitol 2 with the water soluble fraction and xylitol 1 with the water soluble fraction, respectively (Fig. 2A). Moreover, the body weight of mice receiving xylitol 2 with the water soluble fraction recovered more rapidly than that of the mice receiving xylitol 1 with the water soluble fraction group (Fig. 2B). The beneficial effect of xylitol is clearly dosage dependent.

### The Effect of Dietary Xylitol with the RG Water Soluble Fraction on Lung Virus Titer

The xylitol 2+ water soluble fraction group received xylitol 2 with the water soluble fraction orally for 5 days prior to virus challenge and for 3 days post virus challenge. The oseltamivir group received phosphate-buffered saline (PBS) orally for 5 days prior to virus challenge and oseltamivir orally for 7 days post challenge. Mice were challenged with 2X LD_50_ of influenza A virus, and lungs was collected 3 days post challenge. The median virus titers in the lungs of mice receiving PBS, oseltamivir and xylitol 2 with water soluble RG fraction were 7.63, 5.93 and 7.28 (log_10_ titers), respectively (Fig. 3). The virus tiers of mice receiving PBS, oseltamivir and xylitol 2 with water soluble RG fraction ranges 7.47–7.65, 4.82–5.98 and 7.22–7.31, respectively (Fig. 3). Therefore, the treatment with xylitol 2 combined with water soluble RG fraction was confirmed to reduce the lung virus titer. This result indicates that dietary xylitol together with the water soluble fraction exerts a protective effect against influenza A virus.

### The Protective Effect as a Function of Treatment Length

**Figure 4:**
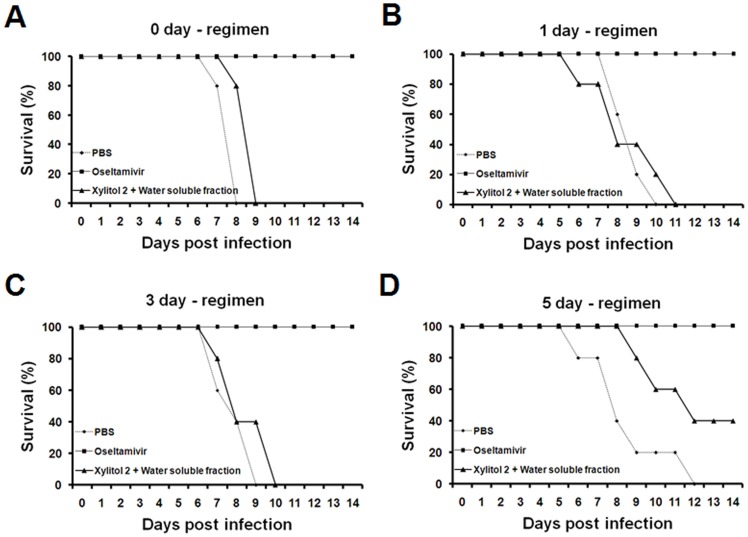
Mouse survival as a function of dosing period before lethal influenza A virus challenge. Mice received PBS and xylitol 2 (33 mg/kg/day) in combination with the water soluble fraction (0.25 mg/kg/day) orally for 0, 1, 3 or 5 days prior to influenza A virus challenge. Details of the administration protocols are presented in Table 2. Panels A, B, C and D are survivals following administration for 0, 1, 3 and 5 days prior to virus challenge. After challenge with 2X LD_50_ of influenza A virus, each regimen was continued for 3 days and survival was monitored for 14 days post challenge. PBS, n = 5; xylitol 2+ water soluble fraction, n = 5; oseltamivir, n = 3.

To investigate the effect of duration of treatment on protection, mice were given xylitol 2 combined with the water soluble fraction for 0, 1, 3 or 5 days prior to virus challenge (Table 2). No protective effect was observed when treatment lasted for 0, 1 or 3 days prior to virus challenge (Fig. 4A, B and C) whereas, 40% of the mice survived after 5 days of treatment (Fig. 4D).

**Table 2:** Administration protocols for the 0, 1, 3 and 5

Regimen	Mouse group	Prior to virus challenge	Post virus challenge
0-day	PBS	–	PBS 200 μl/day (for 3 days)
	Oseltamivir	–	Oseltamivir 10 mg/kg/day (for 7 days)
	Xylitol 2+water soluble fraction	–	33 mg/kg/day +0.25 mg/kg/day (for 3 days)
1-day	PBS	PBS 200 μl/day (for 1 day)	PBS 200 μl/day (for 3 days)
	Oseltamivir	PBS 200 μl/day (for 1 day)	Oseltamivir 10 mg/kg/day (for 7 days)
	Xylitol 2+water soluble fraction	33 mg/kg/day +0.25 mg/kg/day (for 1 day)	33 mg/kg/day +0.25 mg/kg/day (for 3 days)
3-day	PBS	PBS 200 μl/day (for 3 days)	PBS 200 μl/day (for 3 days)
	Oseltamivir	PBS 200 μl/day (for 3 days)	Oseltamivir 10 mg/kg/day (for 7 days)
	Xylitol 2+water soluble fraction	33 mg/kg/day +0.25 mg/kg/day (for 3 days)	33 mg/kg/day +0.25 mg/kg/day (for 3 days)
5-day	PBS	PBS 200 μl/day (for 5 days)	PBS 200 μl/day (for 3 days)
	Oseltamivir	PBS 200 μl/day (for 5 days)	Oseltamivir 10 mg/kg/day (for 7 days)
	Xylitol 2+water soluble fraction	33 mg/kg/day +0.25 mg/kg/day (for 5 days)	33 mg/kg/day +0.25 mg/kg/day (for 3 days)

The results as a function of treatment period are presented in Fig. 4.

## Discussion

Much attention has been paid to the local inhibitory effect of xylitol on the growth of cavity-causing bacteria in the mouth. However, there is some evidence that dietary xylitol induces systemic changes. It enhanced levels of serum calcium and serum alkaline phosphatase activity and bone density in a rat model [18], and prolonged the survival of rats in pneumococcal-induced sepsis [19]. In the present study we prepared various fractions of RG and found that all of them synergized with xylitol in increasing the survival of mice challenged with influenza virus (Fig. 1). This implies that the synergism with xylitol need not be limited to RG constituents, since saponins are found in a variety of plants [20], and polysaccharide is the main constituent of the plant cell wall [21], and presumably these would also synergize with xylitol. RG polysaccharide is known to have anti-hyperlipodemic, immunomodulatory and anticancer effects, and RG saponin has anticancer and antineoplastic effects [22]–[26]. Whether xylitol and RGs have beneficial synergistic effects in other diseases remains to be examined.

Xylitol 2 plus the water soluble RG fraction had the greatest effect when given for 5 days prior to virus infection (Fig. 1 and 2). As shown in Fig. 2B, there was little loss of body weight when oseltamivir, a neuraminidase inhibitor, was given post virus challenge. In contrast, the mice receiving xylitol 2 plus the water soluble fraction suffered a rapid reduction in body weight post virus challenge (Fig. 2B) although all survived. This result indicates that the protective mechanism of xylitol and water soluble fraction is different from that of oseltamivir.

There was variation of survival rate in mice receiving xylitol 2 in combination with water soluble fraction: 60% (Fig. 1), 100% (Fig. 2A) and 40% (Fig. 4D) of mice survived following challenge with 2X LD_50_ of influenza virus. As shown in Fig. 2B, the challenge with 2X LD_50_ of virus reduced the body weights of mice receiving xylitol 2 with water soluble fraction by 74% of their initial body weights. This result indicates that the mice maintain their life by a narrow margin when the mice were challenged with 2X LD_50_ of virus. The challenge with 2X LD_50_ of influenza virus showed the synergistic effects of treatment of xylitol along with water soluble RG fraction effectively (Fig. 1 and 2). However, the experimental design, which reduces the body weight of mice treated with xylitol combined with water soluble RG fraction to the borderline of death, is thought to result in the variation of mouse survival.

There was no effect when xylitol plus the water soluble fraction was given only after virus challenge (see 0-day regimen, Fig. 4A) and protection was only observed with 5-day treatments prior to challenge, not when it was given for 1 or 3 days prior to challenge (Fig. 4B, C and D). Therefore, it is unlikely that this treatment affects influenza virus directly. Numerous studies have shown that RGs have immunomodulatory effects [22]–[26]. Moreover, we found previously that dietary RGs given for 14 days prior to influenza virus infection reduced influenza-induced symptoms by acting on dendritic cells (tipDCs) producing tumor necrosis factor alpha (TNF-α)/inducible nitric oxide synthase (iNOS) in the lungs [12]. In this study, similarly, dietary xylitol with water soluble fraction was confirmed to reduce the level of tipDC in the bronchoalveolar lavage (BAL) fluid following 2X LD_50_ of influenza virus challenge (Fig. S1), suggesting that the xylitol with water soluble fraction contributes to the reduction of influenza-induced symptoms by modulating the immune system. Moreover, it has been proposed that dietary xylitol enhances the activity of neutrophils in rats [19]. Taken together, these findings suggest that the enhanced survival (Fig. 1 and 2) and reduced viral loads in mouse lungs (Fig. 3) after lethal influenza virus infection are associated with immunomodulatory effects of xylitol and RGs.

The aim of this study was to investigate whether dietary xylitol can modulate the symptoms induced by influenza virus infection. We found no ameliorating effect when xylitol or RGs were administered separately, only when they were given together for 5 days. Since a 14 day-regimen is required for RGs on their own to exert a protective effect on influenza virus infection [11], [12] our results show that the treatment period can be markedly reduced if xylitol is administered along with RGs.

The genome of influenza virus mutates spontaneously, and some mutations confer resistance to anti-influenza virus agents. A recent report indicated that 27% of seasonal influenza A virus particles (H1N1) are resistant to oseltamivir [27]. Similarly all influenza A viruses (H3N2) and the 2009 Mexican flu pandemic influenza A virus (H1N1) were resistant to adamantanes [9]. The Centers for Disease Control and Prevention (CDC) has stated that amantadine and rimantadine are not recommended for antiviral treatment or chemoprophylaxis of the currently circulating influenza A virus strains [28]. Recent evidence clearly indicates that resistance to anti-influenza agents gradually increases among prevalent influenza viruses. Hence there is a great need for new strategies for preventing and controlling influenza virus infections, and further analysis of the protective effect of xylitol could lead to new possibilities for controlling influenza virus infections.

## Materials and Methods

### Ethics

All animal experiments were treated in accordance with the guideline of Institutional Animal Care and Use Committee, Chung-Ang University IACUC, and the protocol was approved by the IACUC (approval no.: 13-0039). Virus challenges in mice were performed under approved anesthesia, and all efforts were made to minimize suffering. Mice were anesthetized intraperitoneally with 10 µl of 4∶1 mixture of Zoletil 50 (Virbac, France) and Rompun (Bayer Animal Health, Germany) for intranasal instillation of virus. The conditions of mice were monitored twice a day. The humane endpoint was used during survival study: mice were euthanized using CO_2_ gas when body weights were reduced to 70% of the starting weights. Mice for survival studies were euthanized using CO_2_ gas and those for virus titration of lung were euthanized by excessive anesthetization (30 µl of 4∶1 mixture of Zoletil 50 and Rompun).

### Preparation of Xylitol

Xylitol was obtained from Sigma (X3375, Sigma, USA); 66 mg of xylitol was dissolved in 10 ml Krebs-Henseleit fluid. Krebs-Henseleit solution contains 6.9 g NaCl, 0.35 g KCl, 0.28 g CaCl_2_, 0.16 g KH_2_PO_4_, 0.2 g MgSO_4,_ 2.09 g NaHCO_3_, and 0.22 g Na-pyruvate per liter [29].

### Preparation of Whole Extract of RG, Water Soluble Fraction, Polysaccharide and Saponin

Whole extract, polysaccharide and saponin of RG were prepared as described previously [12]. To obtain total RG, total extract and RG fractions, four-year-old RG was disrupted with a crusher. Total RG extract was obtained by hot water extraction. To obtain the water soluble fraction, the total RG extract was clarified by centrifugation at 12,000×*g* for 10 min and dialyzed overnight against distilled water (DW) at 4°C. To obtain the polysaccharide fraction, the water soluble fraction was loaded onto a diethylaminoethyl cellulose (DEAE) sepharose CL-6B column (8 mL resin, GE Healthcare, U.S.A.) [30]. Material bound to the DEAE resin was eluted by addition of DW containing 0.25, 0.5 and 0.75 M NaCl. The elutes were dialyzed against DW. Carbohydrate content was measured by the phenol-sulfuric acid method [31], and the polysaccharide finally recovered was confirmed to be almost 100% pure. To obtain RG saponin, whole RG extract was set to 70% saturation with ammonium sulfate and the precipitate was dialyzed against DW, freeze-dried and extracted with methanol. The saponin contained in the methanol fraction was then precipitated by addition of diethyl ether. The purity of the saponin was confirmed by thin layer chromatography (TLC), using ginseng saponin (Ambo Institute, South Korea) as standard. Extracts and fractions were freeze-dried and resuspended in PBS.

### Preparation of Influenza A Virus

Influenza A/PR/8/34 virus (H1N1 subtype) was obtained from egg allantoic fluid. Influenza A virus was propagated in 11-day-old fertilized chicken eggs at 37°C for 48 h. The allantoic fluid gestated was collected and clarified 682×*g* for 10 min and filtered using a 0.22 µm syringe filter [11]. The 50% lethal dose (LD_50_) of the virus stock was determined as reported previously [32].

### Mouse Experiments

6-week-old female Balb/c mice (Orient Bio Inc., South Korea) were acclimatized for 1 week. Thirteen kinds of mouse treatment group were designed, each consisting of five mice (Table 1). Mice received xylitol at a dosage of 3.3 mg/kg/day (xylitol 1) or 33 mg/kg/day (xylitol 2) for 5 days prior to virus challenge, and this was combined with RGs at 0.25 mg/kg/day where indicated. Mouse receiving both xyitol and RGs received the RGs first, followed 8 h later by the xylitol. The PBS control mice received 200 μl PBS/day. The oseltamivir group received PBS orally for 5 days prior to virus challenge followed by oseltamivir (10 mg/kg/day) for 7 days after the virus challenge (Table 1). Mice receiving PBS, xylitol, RGs and xylitol in combination with RGs were exposed to 50 μl of influenza A virus (2X LD_50_ of virus) by intranasal instillation, and the relevant regimens were continued for 3 days post virus challenge. Survival and body weight were monitored for 14 days after virus challenge.

To investigate the effect of dosing period of xylitol 2 in combination with the water soluble fraction, mice received PBS or xylitol 2 along with water soluble fraction orally for 0, 1, 3 or 5 days prior to virus challenge (Table 2) and the treatments were continued for a further 3 days (Table 2). The oseltamivir group received PBS orally for 0, 1, 3 or 5 days prior to virus challenge and for 7 days post virus challenge (Table 2). Survival and body weight were monitored for 14 days after virus challenge.

### Analysis of Viral Titers in the Lung

Viral titers in the mouse lungs were measured as described previously [11]. Mice received PBS, oseltamivir or xylitol along with RG water soluble fraction orally for 5 days and were challenged with 2X LD_50_ of influenza A virus. Whole lungs were collected on day 3 post virus challenge, homogenized with a Dounce homogenizer and centrifuged at 12,000xg for 10 min. Madin-Darby canine kidney (MDCK) [11] cells were cultured in DMEM (GenDEPOT, USA) containing 10% FBS (HyClone, USA) and 1% penicillin/streptomycin (Invitrogen, USA). MDCK cells were seeded into 6-well tissue culture plates at a density of 1×10^5^ cells per well and incubated for 2 day for the virus titration. Thereafter the cells were incubated with rocking for 1 h with the various whole lung supernatants diluted 1∶10,000 or 1∶100,000 with DMEM without FBS. After removal of the supernatants, the cells were incubated in DMEM containing 1% low melting point agarose (Lonza, Inc., USA) and 1 µg/ml trypsin (Invitrogen, USA) for 2 days. They were then stained with 1% crystal violet and 20% methanol in PBS, and plaques were observed.

### Statistical Analysis

The statistical significance of differences between groups was determined by two-tailed Student's *t*-tests. *P*-values less than 0.05 were considered statistically significant.

## Supporting Information

Figure S1 TipDC levels in mice BAL fluids following influenza A virus challenge. Mice received PBS, oseltamivir or xylitol 2 with water soluble fraction orally for 5 days prior to virus challenge and 3 days post virus challenge. After 2X LD_50_ of virus challenge, BAL fluid cells were collected on day 1 post virus challenge and stained with anti-CD11b and -Ly6c antibodies. The proportions of tipDCs among BAL fluid cells were analyzed by scoring CD11b^+^ Ly6c^+^ cells by flow cytometry. Panel A shows representative plots of each mouse group, and panel B shows tipDC levels. The center line of the box represents the median, and the top (Q3) and bottom (Q1), the 75^th^ and 25^th^ percentiles, respectively. The top and bottom whiskers represent outliers. The numbers in parenthesis are median values. PBS, n = 8; oseltamivir, n = 8; xylitol 2+ water soluble fraction, n = 8.
(TIF)
