# Supplementary figures and images for: Protective Effect of Dietary Xylitol on Influenza A Virus Infection
Source: PLoS One. 2014 Jan 2;9(1):e84633. doi: 10.1371/journal.pone.0084633 (PMC3879333; doi:10.1371/journal.pone.0084633)

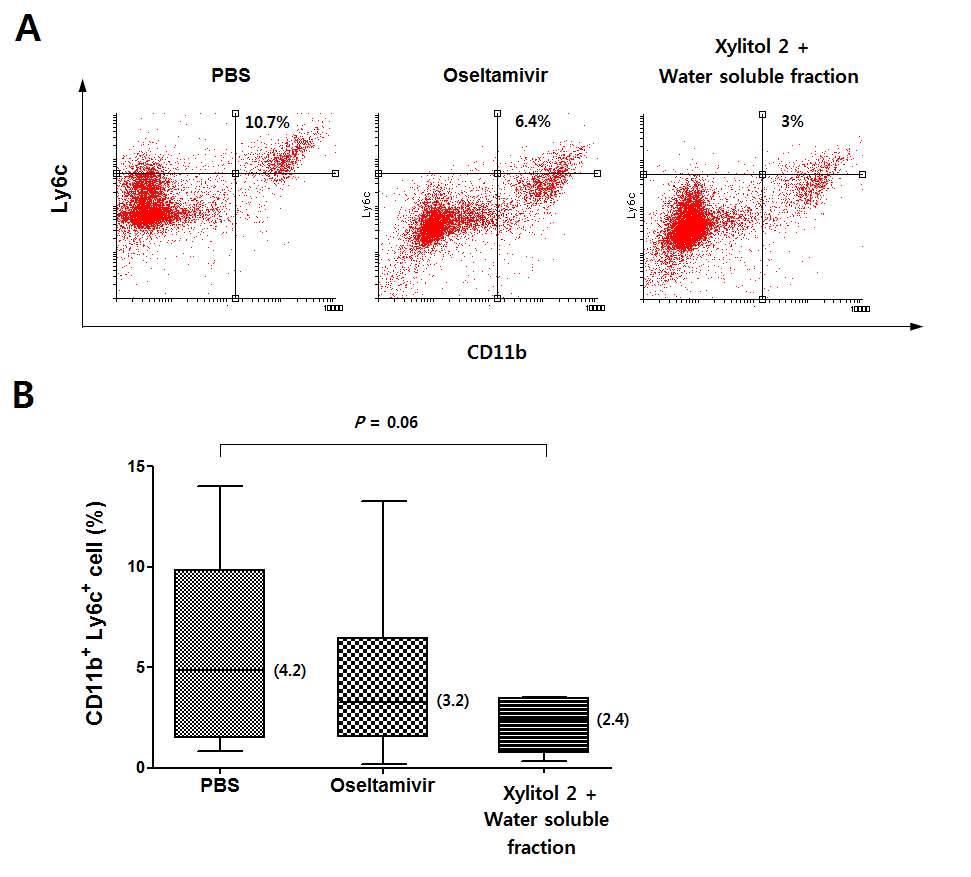

Supplement: Figure S1 — TipDC levels in mice BAL fluids following influenza A virus challenge. Mice received PBS, oseltamivir or xylitol 2 with water soluble fraction orally for 5 days prior to virus challenge and 3 days post virus challenge. After 2X LD50 of virus challenge, BAL fluid cells were collected on day 1 post virus challenge and stained with anti-CD11b and -Ly6c antibodies. The proportions of tipDCs among BAL fluid cells were analyzed by scoring CD11b+ Ly6c+ cells by flow cytometry. Panel A shows representative plots of each mouse group, and panel B shows tipDC levels. The center line of the box represents the median, and the top (Q3) and bottom (Q1), the 75th and 25th percentiles, respectively. The top and bottom whiskers represent outliers. The numbers in parenthesis are median values. PBS, n = 8; oseltamivir, n = 8; xylitol 2+ water soluble fraction, n = 8. (TIF) [file pone.0084633.s001.tif]
